# Supplementary material for: A Single Standard to Determine Multi-Components Method Coupled with Chemometric Methods for the Quantification, Evaluation and Classification of Notopterygii Rhizoma et Radix from Different Regions
Source: Molecules. 2019 Oct 3;24(19):3574. doi: 10.3390/molecules24193574 (PMC6804041; doi:10.3390/molecules24193574)
Supplement: Supplementary file 1 [file molecules-24-03574-s001.pdf]

Supporting documents

A single standard to determine multi-components method coupled with chemometric methods for the quantification, evaluation and classification of *Notopterygii* Rhizoma et Radix from different regions

Xie-an Yu[1,2], Jin Li[1,†], John Teye Azietaku[1,2], Wei Liu[1,2], Jun He[1,2*], Yan-xu Chang[1,2*]

[1]Tianjin State Key Laboratory of Modern Chinese Medicine, Tianjin University of Traditional Chinese Medicine, Tianjin 300193, China
[2]Tianjin Key Laboratory of Phytochemistry and Pharmaceutical Analysis, Tianjin University of Traditional Chinese Medicine, Tianjin 300193, China
*Correspondence: hejun673@163.com (J.H.); tcmcyx@tjutcm.edu.cn (Y.C.);
† These authors contributed equally to this work.
Tel./Fax: + 86-22-59596163 (J.H. &Y.C.)

Table1. Data for the calibration curves, LODs, LOQs, R_f and repeatability using ES method (n = 6).

Compounds	Regressive equation	Line range (μg mL^{-1})	R	R_f	LOD (μg mL^{-1})	LOQ (μg mL^{-1})	Repeatability
							RSD(%)
Chlorogenic acid	Y=8509.311X-113.31	0.2-50	0.9999	1	0.03	0.1	1.25
p-Coumaric acid	Y=7248.429X-293.686	0.2-25	0.9996	0.783	0.06	0.2	2.39
Scopoletin	Y=9064.661X+291.485	0.2-25	0.9998	2.234	0.06	0.2	1.81
Ferulic acid	Y=11890.98X-1847.27	0.4-100	0.9999	1.291	0.06	0.2	2.36
Coumarin	Y=2289.637X+10.744	0.4-50	0.9999	0.528	0.15	0.4	2.69
Nodakenin	Y=8779.59X-291.147	0.4-100	0.9999	1.028	0.035	0.1	1.89
Bergaptol	Y=5205.188X-195.825	0.2-50	0.9999	1.141	0.06	0.2	2.16
Psoralen	Y=5343.163X-92.878	0.16-20	0.9999	1.163	0.05	0.15	4.09
Angelicin	Y=2935.177X-2.095	0.16-20	0.9997	0.672	0.05	0.15	3.06
8-Methoxypsoralen	Y=2590.638X+61.142	0.1-25	0.9999	0.628	0.06	0.2	2.86
Bergapten	Y=5957.552X-68.930	0.1-25	0.9999	1.329	0.03	0.1	1.63
Diosmetin	Y=10084.27X-186.211	0.08-20	0.9998	2.187	0.02	0.08	3.17
Byakangelico	Y=3351.521X-66.420	0.2-50	0.9998	0.751	0.06	0.2	1.16
Imperatorin	Y=2247.772X-112.236	0.4-100	0.9999	0.5	0.15	0.4	2.02
Phelloptorin	Y=3329.094X+50.928	0.1-25	0.9999	0.791	0.03	0.1	2.63
Osthole	Y=8868.786X+12.322	0.32-80	0.9999	2.035	0.03	0.1	2.61
Notopterol	Y=4214.462X+252.086	0.4-100	0.9999	1	0.06	0.2	1.77
Isoimperatorin	Y=2403.511X-29.034	0.4-100	0.9999	0.547	0.06	0.2	1.67
Praeruptorin A	Y=5169.775X+161.942	0.15-25	0.9999	1.272	0.05	0.15	2.63

Table 2. Intraday and interday precision of the 19 compounds using ES method (n = 6).

Compounds	Concentration (μg mL^{-1})	Intra-day ES		Inter-day ES	
		Accuracy(%)	RSD(%)	Accuracy (%)	RSD(%)
	0.3	101	3.76	102	2.71
Chlorogenic acid	3	96.1	2.01	97.0	2.19
	30	105	2.42	99.9	3.90
	0.25	104	2.20	100	4.73
p-Coumaric acid	2.5	100	2.05	101	2.65
	25	100	3.19	100	3.47
	0.25	104	3.64	102	3.64
Scopoletin	2.5	103	3.05	103	2.87
	25	103	2.33	102	2.70
	0.6	102	1.99	103	1.96
Ferulic acid	6	104	3.13	102	3.19
	60	104	2.99	104	2.96
	0.4	104	2.83	101	3.59
Coumarin	4	104	3.18	104	2.70
	40	103	3.52	103	2.92
	0.6	104	2.60	103	2.02
Nodakenin	6	104	1.12	104	1.32
	60	104	3.58	103	2.91
	0.4	97.7	2.05	100	3.41
Bergaptol	4	104	2.77	103	3.05
	40	104	1.53	101	4.25
Psoralen	0.2	96.7	2.18	99.9	3.40

	2	103	2.23	102	2.83
	20	107	1.25	104	2.46
	0.2	100	4.65	100	4.56
Angelicin	2	99.4	5.12	102	3.87
	20	105	1.79	101	4.33
	0.2	98.6	2.05	100	3.61
8-Methoxypsoralen	2	102	3.23	101	3.54
	20	99.4	4.08	102	3.96
	0.2	104	2.23	103	2.85
Bergapten	2	104	4.07	101	4.39
	20	102	1.34	99.9	4.44
	0.2	103	3.13	102	3.08
Diosmetin	2	106	1.38	105	2.10
	20	107	1.05	103	4.67
	0.4	104	4.31	101	3.76
Byakangelico	4	104	1.86	103	2.24
	40	101	1.62	103	2.71
	0.8	105	0.91	104	1.92
Imperatorin	8	105	0.95	104	1.37
	80	103	1.03	104	2.00
	0.2	96.8	1.78	98.8	3.68
Phelloptorin	2	105	3.06	102	3.84
	20	98.3	5.12	101	5.03
	0.5	101	1.13	101	2.08
Osthole	5	103	1.82	103	2.14
	50	104	2.43	104	1.93

	0.6	102	2.45	101	2.82
Notopterol	6	97.7	4.57	96.8	2.90
	60	101	4.54	97.5	4.19
	0.6	102	3.43	101	2.86
Isoimperatorin	6	104	1.66	101	3.13
	60	99.5	2.95	102	4.42
	0.2	101	5.29	100	3.77
Praeruptorin A	2	97.3	3.06	99.2	3.04
	20	99.2	3.80	100	3.99

Table 3. The stability of the 19 compounds using ES method (n=6).

Compounds	Concentration (μg mL^{-1})	Stability	
		Accuracy (%)	RSD (%)
		99.5	4.76
Chlorogenic acid	3	95.7	2.63
	30	93.9	1.69
	0.25	106	2.22
p-Coumaric acid	2.5	98.8	2.89
	25	102	5.65
	0.25	97.5	6.18
Scopoletin	2.5	103	4.31
	25	106	3.36
Ferulic acid	0.6	102	3.39
	6	107	2.77

	60	110	5.42
	0.4	101	5.11
Coumarin	4	105	5.47
	40	108	3.32
	0.6	101	2.76
Nodakenin	6	106	3.59
	60	106	3.13
	0.4	104	1.68
Bergaptol	4	104	3.19
	40	108	3.28
	0.2	102	4.41
Psoralen	2	104	5.55
	20	106	5.07
	0.2	96.8	4.65
Angelicin	2	104	4.13
	20	103	5.21
	0.2	97.0	4.44
8-Methoxypsoralen	2	106	4.77
	20	106	3.79
	0.2	103	4.71
Bergapten	2	108	2.80
	20	108	5.02
	0.2	105	3.94
Diosmetin	2	108	1.65
	20	113	3.75
Byakangelico	0.4	102	6.64

	4	109	3.27
	40	105	2.94
	0.8	102	3.56
Imperatorin	8	108	4.26
	80	104	1.29
	0.2	96.0	4.54
Phelloptorin	2	102	2.95
	20	106	1.59
	0.5	103	2.69
Osthole	5	107	5.18
	50	103	4.83
	0.6	97.2	1.89
Notopterol	6	96.0	3.43
	60	94.6	6.10
	0.6	100	2.97
Isoimperatorin	6	101	3.76
	60	105	5.44
	0.2	94.7	5.01
Praeruptorin A	2	99.6	3.58
	20	98.7	3.33

Table 4. The recovery of the 19 compounds using ES method (n=6).

Compounds	Origin	Added	ES	
			Recovery(%)	RSD(%)
Chlorogenic acid	3.47	2.80	99.3	3.04

		3.50	106	4.41
		4.20	98.7	4.20
		0.80	102	2.82
p-Coumaric acid	1.09	1.00	106	3.49
		1.20	105	5.96
		0.16	94.8	4.76
Scopoletin	0.22	0.20	94.5	2.23
		0.24	99.5	3.13
		6.40	103	5.80
Ferulic acid	7.97	8.00	104	6.46
		9.60	108	3.44
		0.24	99.4	4.00
Coumarin	0.31	0.30	103	2.90
		0.36	96.4	6.41
		6.40	103	3.07
Nodakenin	7.93	8.00	109	6.27
		9.60	108	4.28
		3.20	109	4.41
Bergaptol	3.95	4.00	102	5.32
		4.80	95.5	3.24
		0.16	102	5.86
Psoralen	0.17	0.20	96.7	6.70
		0.24	102	3.44
		0.32	96.0	5.49
Angelicin	0.37	0.40	102	2.64
		0.48	95.2	1.04

		0.32	95.3	1.24
8-Methoxypsoralen	0.40	0.40	101	5.07
		0.48	94.8	3.75
		0.16	102	5.55
Bergapten	0.20	0.20	108	2.25
		0.24	107	5.82
		0.03	100	6.27
Diosmetin	0.04	0.04	98.1	6.77
		0.05	97.1	3.64
		2.40	101	5.49
Byakangelico	2.68	3.00	105	4.71
		3.60	105	6.42
		0.24	99.6	5.03
Imperatorin	0.30	0.30	97.1	0.65
		0.36	95.2	6.84
		0.12	98.8	4.20
Phelloptorin	0.13	0.15	109	5.62
		0.18	103	5.22
		24.00	105	2.42
Osthole	27.40	30.00	109	5.11
		36.00	89.1	7.79
		40.00	93.7	6.55
Notopterol	48.42	50.00	95.4	3.39
		60.00	97.2	4.49
Isoimperatorin	22.93	16.00	102	4.18
		20.00	106	5.73

		24.00	109	7.78
		0.24	101	5.07
Praeruptorin A	0.32	0.30	107	4.14
		0.40	109	5.42

Table 5. Contents of 19 compounds in different sample using ES method.

Contant (mg g^{-1}) EX	compounds																		
	Chloro genic acid	p-Coum aric acid	Scopo letin	Ferulic acid	Coum arin	Noda kenin	Berg aptol	Psora len	Ange licin	8-Meth Oxyps oralen	Berga pten	Dios metin	Byak Ange lico	Impera torin	Phello ptorin	Osth ole	Noto pterol	Isoim Perat orin	Praeru ptorin A
1Sichuan(chengdu)	0.55	0.19	0.00	0.70	0.02	1.79	0.15	0.02	0.02	0.02	0.01	0.00	0.48	0.00	0.12	4.53	2.54	1.43	0.13
2Sishuan(meishan)	3.01	0.68	0.03	2.32	0.03	1.53	0.22	0.08	0.06	0.10	0.06	0.00	1.22	0.05	0.04	7.09	9.23	4.30	0.00
3Sichuan(leshan)	0.33	0.09	0.02	0.74	0.00	0.81	0.39	0.02	0.02	0.06	0.03	0.00	0.24	0.05	0.03	2.62	4.57	2.24	0.03
4Sichuan(ya'an)	0.25	0.20	0.03	0.62	0.00	0.74	0.13	0.02	0.06	0.04	0.01	0.29	0.43	0.37	0.15	2.80	5.05	3.39	0.02
5Sichuan(guanghan)	0.78	0.08	0.00	0.81	0.07	0.95	0.12	0.00	0.03	0.08	0.16	0.02	0.66	101.46	0.04	0.30	1.20	22.65	0.47
6Sichuan(mianyang)	0.76	0.13	0.03	1.06	0.02	5.53	0.32	0.04	0.09	0.11	0.17	2.95	1.36	2.46	0.21	2.18	3.84	4.48	0.00
7Sichuan(luzhou)	0.24	0.29	0.02	0.75	0.02	1.34	0.32	0.03	0.02	0.06	0.03	0.02	0.49	0.31	0.04	4.82	6.19	3.96	0.02
8Shanxi	0.15	0.51	0.00	0.04	0.00	0.16	0.15	0.04	0.49	0.62	0.04	0.00	0.10	0.20	0.11	0.16	3.79	0.54	1.48
9Gansu	0.70	0.16	0.03	0.80	0.02	1.22	0.20	0.04	0.02	0.06	0.04	0.02	0.42	0.11	0.03	2.27	3.78	2.14	0.03
10Gansu	1.06	0.05	0.08	0.62	0.04	29.16	1.47	0.03	0.20	0.15	0.14	0.03	3.11	0.12	0.63	0.06	0.19	30.85	0.00
11Sichuan(yibin)	1.73	0.48	0.04	1.12	0.01	0.77	0.14	0.05	0.13	0.11	0.02	0.00	0.72	0.11	0.03	4.51	5.21	1.97	0.00
12Sichuan(xinjin)	0.10	0.03	0.00	0.28	0.01	0.36	0.61	0.02	0.01	0.07	0.02	0.27	0.07	0.14	0.04	0.59	2.32	3.26	0.00
13Sichuan(bazhong)	0.17	0.04	0.00	0.46	0.03	22.73	2.78	0.01	0.13	0.14	0.15	5.64	3.05	0.34	0.58	0.44	0.84	22.41	0.06
14Sichuan(neijiang)	0.34	0.14	0.00	0.82	0.00	0.69	0.09	0.04	0.02	0.10	0.03	0.02	0.60	0.03	0.03	3.21	5.82	1.20	0.25
15Sichuan(ziyang)	1.26	0.40	0.03	1.00	0.01	0.79	0.33	0.03	0.06	0.09	0.03	0.00	0.67	0.22	0.03	4.02	6.06	2.24	0.00

16Yunnan(kunming)	1.56	0.13	0.02	0.57	0.01	27.56	0.78	0.03	0.27	0.12	0.27	0.27	2.18	0.50	0.38	0.05	0.19	25.92	0.00
17Qinhai	2.71	0.23	0.03	0.86	0.01	16.29	0.21	0.03	0.15	0.07	0.22	0.01	0.33	0.00	0.99	1.10	1.82	10.22	0.00
18Sichuan	0.87	0.22	0.05	0.81	0.02	4.97	0.59	0.08	0.04	0.23	0.03	0.01	0.28	4.58	0.09	6.23	0.87	3.91	0.38
19Sichuan	0.32	0.21	0.04	0.80	0.01	4.35	1.65	0.02	0.04	0.11	0.00	0.03	0.35	6.76	0.06	4.56	2.78	4.85	0.00
20Sichuan	0.33	0.29	0.00	0.00	0.00	0.10	0.30	0.03	0.02	0.22	0.01	0.00	0.03	0.03	0.00	0.14	0.55	2.81	0.78
21Sichuan	0.37	0.13	0.05	0.75	0.01	1.34	0.76	0.07	0.04	0.16	0.03	0.24	0.33	0.40	0.04	3.19	0.61	2.12	0.00
22Shandong(taishan)	1.50	0.24	0.02	1.03	0.07	1.21	0.17	0.07	0.06	0.16	0.06	0.00	0.28	0.44	0.02	2.35	2.01	0.88	0.89
23Neimeng	0.16	0.94	0.03	0.10	0.03	0.14	0.16	0.05	1.71	0.27	0.06	0.01	0.10	0.40	0.33	0.16	1.43	3.02	2.99
24Gansu	0.31	0.06	0.02	0.51	0.03	8.40	0.37	0.00	0.08	0.07	0.04	0.00	0.45	0.08	0.06	0.65	0.81	6.22	0.00
25Gansu	0.79	0.11	0.00	1.10	0.01	14.77	0.78	0.03	0.20	0.08	0.09	0.03	1.51	0.00	0.14	2.06	2.36	12.09	0.00
26（Anhui）	0.15	0.12	0.02	0.39	0.01	0.45	0.41	0.05	0.01	0.53	0.06	0.01	0.17	0.60	0.02	1.87	4.26	2.61	4.67
27Sichuan	0.29	0.68	0.00	2.17	0.04	1.02	0.43	0.03	0.02	0.06	0.05	0.00	0.63	0.00	0.04	4.67	7.04	11.17	0.00
28Sichuan	0.11	0.06	0.00	0.48	0.01	0.55	0.23	0.00	0.01	0.02	0.04	0.01	0.14	0.10	0.00	1.39	0.73	0.85	0.02
29Tianjin	0.19	0.38	0.02	0.57	0.01	0.59	0.17	0.03	0.02	0.08	0.00	0.01	0.55	0.03	0.03	3.37	0.54	0.84	0.00
30Shanxi	0.48	0.06	0.00	1.18	0.00	31.82	2.93	0.02	0.13	0.16	0.31	0.03	0.53	0.27	0.54	0.04	3.27	0.04	0.00
31Shanxi	1.65	0.20	0.00	0.98	0.08	48.99	2.97	0.03	0.25	0.15	0.29	0.06	3.22	0.40	0.69	0.01	6.37	0.03	0.00
32Shanxi	0.73	0.14	0.00	1.35	0.00	70.31	3.76	0.03	0.30	0.36	0.24	0.05	2.19	0.63	0.64	0.00	7.38	0.06	0.00
